# Use of sodium fluorescein in skull base tumors

**DOI:** 10.4103/2152-7806.72247

**Published:** 2010-10-30

**Authors:** Carlos Eduardo da Silva, Jefferson Luis Braga da Silva, Vinicius Duval da Silva

**Affiliations:** Department of Neurosurgery and Skull Base Surgery, Hospital Ernesto Dornelles, Porto Alegre/RS, Brazil; 1 Service of Hand Surgery and Reconstructive Microsurgery, Hospital São Lucas, Brazil; 2 Department of Pathology and Radiation, FAMED, Pontifical Catholic University of Rio Grande do Sul, Porto Alegre/RS, Brazil

**Keywords:** Cranial base tumors, fluorescence-guided surgery, sodium fluorescein

## Abstract

**Objective:**

The authors present this study using sodium fluorescein (SF) to enhance skull base tumors by performing a quantitative digital analysis of tumor enhancement. The purpose of this study is to observe the grade of SF enhancement by the tumors.

**Methods:**

A prospective experiment within-subjects study design was performed which included six patients with skull base lesions. Digital pictures were taken before and after the SF systemic injection, using the same light source of the microsurgical field. The pictures were analyzed by computer software which calculated the wavelength (WL) of the SF pre- and post-injection.

**Results:**

The group of tumors was as follows: one vestibular schwannoma, three meningiomas, one craniopharyngioma and one pituitary adenoma. The SF enhancement in all tumors was strongly positive. The digital analysis of the pictures, considering the SF WL pre- and post-injection, presented *P* = 0.028 (Wilcoxon *T* test).

**Conclusions:**

The enhancement of the tumors by SF was consistent and evident. The introductory results suggest the possibility of using SF as an adjuvant tool for the skull base surgery. Further studies should test the clinical application of the SF in skull base tumors.

## INTRODUCTION

The skull base tumors are neurosurgical challenges due to neurovascular structures involved in the treatment of these lesions. Numerous surgical techniques and equipments have been developed to access the deep anatomical sites in order to improve the outcomes of such complex lesions.

Sodium fluorescein (SF) was first used for the identification of different types of brain tumors in 1948.[20] Since then, the use of SF and others fluorescent markers has been described in the literature especially dealing with glioblastoma multiform resection.[16,18,19,32,35,37] Nevertheless, SF was not portrayed as an adjuvant for the surgical resection of skull base lesions.

The authors present this introductory experiment within-subjects study using SF in skull base tumors, by performing a quantitative digital analysis of the tumor enhancement by the substance. The application intends to observe the initial results of the skull base tumor enhancement by the SF.

## METHODS

A prospective experiment within-subjects study design was performed, which included six patients with skull base lesions, who were operated between December 2008 and February 2009. The inclusion criteria were patients presenting with tumors located in the anterior, medial or posterior cranial base. The patients were informed about the transoperative experimental use of SF and written consent was obtained before the procedure.

The initial dissections were performed, and after the exposure of the tumors and their relative positioning within cranial nerves and vascular structures, a digital photo was first manually obtained using the optical lens of a microscope. The digital camera used was SONY, model DSC-W90, 8.1 megapixels, with macroactivation on and internal flash off. The light source of the pictures was the same as the microsurgical field, capturing images visualized by the surgeon at the microscope without any special filters.

A dose of 1 g of the SF 20% was injected into a peripheral vein. The second picture was taken 10 minutes after the SF injection, using the same technique described previously.

The pictures were saved in JPEG format with minimal compression and divided into two groups composed of SF pre- and post-injection images. The images were analyzed by the IMAGE PRO PLUS 4.5.1 program (Media Cybernetics, Silver Spring, MD, USA). First, the SF post-injection image was submitted for program analysis. The area of interest was defined using a rectangular frame with the tumor in central position and the surrounding neurovascular structures. Manual selection of colors was performed using level 5 of sensitivity (maximum level, range 1–5). Red color was defined to highlight the wavelength (WL) of the SF in the picture. After the area of enhancement by the SF was defined by the program, it was saved and the program calculated the total area of the picture presenting the SF WL. The absolute value obtained by such statistical analysis of the program was saved in an Excel (Microsoft, Redmond, WA, USA) spreadsheet. The SF pre-injection image of the same case was later analyzed. The same rectangular frame was applied around the tumor and neurovascular structures without SF. The specific SF WL of the post-injection picture recorded by the program was applied in the same selected area of the pre-injection picture, and the program calculated the area presenting the SF WL. The data were saved for statistical analysis in the Excel database.[12,25]

The nonparametrical Wilcoxon test was used for the statistical analysis comparing the values obtained in the two groups composed of the SF WL pre and post-injection pictures.

## RESULTS

The group of six tumors was as follows: one vestibular schwannoma, three meningiomas, one craniopharyngioma and one pituitary adenoma. The meningiomas were located in the sphenoid wing, petroclival and clinoidal regions.

**Figure 1:**
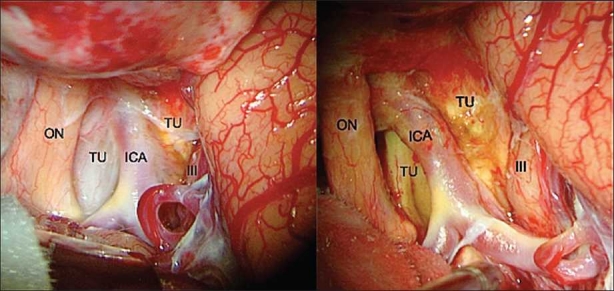
Craniopharyngioma – Intraoperative microsurgical view of the right optic nerve, internal carotid artery and their relation to the tumor. Left: pre SF injection; right: post SF injection (ON: optic nerve; ICA: internal carotid artery; TU: tumor; III: third nerve)

**Figure 2:**
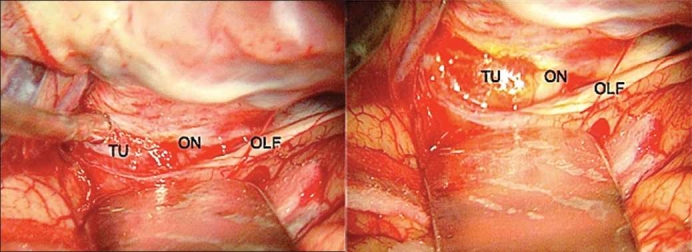
Clinoid meningioma – Intraoperative photograph of the left optic nerve, olfactory nerve and their relation to the anterior clinoid meningioma. Left: pre SF injection; right: post SF injection (ON: optic nerve; TU: tumor; OLF: olfactory nerve)

Table 1 presents the values of the area measured by the IMAGE PRO PLUS program, with the corresponding WL of the SF. The wide range of the simple arithmetical sum of the area of the SF WL probably occurred as a result of the variability of the light during the manual pictures at the ocular lens of the microscope. Such methodological aspect was sustained because the authors would like to capture the image in a real fashion, in order to reproduce the variability of illumination observed during the surgeries. Figures 1 and 2 illustrate the grade of SF enhancement observed at the surgical microscope.

**Table 1:** Area of SF WL measured by digital program

Tumor	SF WL area pre-I	SF WL area post-I
Craniopharyngioma	14.82	63,580.00
Vestibular Schwann	1487.00	107,874.00
Pituitary adenoma	99,865.00	140,639.00
SW meningioma	6,496.00	22,373.00
Clinoid meningioma	5,243.00	114,175.00
PC meningioma	0.37	21.60

*P* = 0.028; SF: sodium fluorescein; WL: wavelength; pre-I: pre-injection; post-I: post-injection; SW: sphenoid wing; PC: petroclival; Schwann: schwannoma

In spite of a few cases, the difference between the groups pre- and post-injection of SF measured by the area with the WL of the SF in the pictures was statistically significant. The Wilcoxon *T* test gave *P* = 0.028.

**Graph 1:**
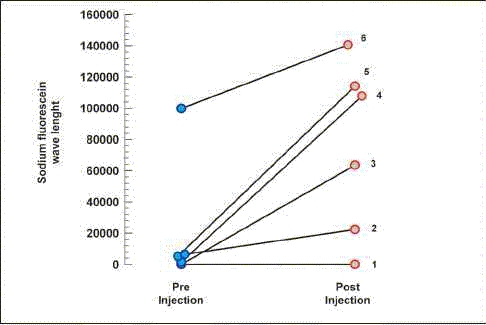
Graph 1: Sodium fluorescein wave length

Graph 1 presents the impact of the caption of the SF by the tumors measuring the SF WL pre- and post-injection.

## DISCUSSION

The impact of the SF in the brain tumor identification was first investigated by Moore *et al*.[20] The SF is used for ophthalmoscopic examinations and several authors tested the applicability of the SF during the surgical removal of glioblastoma multiform.[16,18,19,32,35] Other fluorescence markers have been used as important tools for improvement of glioma resections.[35]

Skull base tumors involve critical neural and vascular structures in most occasions. The surgical technique advances in cranial base surgery promote a progressive improvement of resections and prognosis of such cases.[1–4,9–11,17,21,27,33,38–40] The concern about the morbidity related to dissections around the cranial nerves and arterial and venous vessels is constant during the approaches.[5,8,22,26,34] Many technological advances have been applied to improve the structure preservation, such as neuronavigational systems, intraoperative neurophysiological monitoring and neurofunctional imaging, but the effective maintenance of vascular and cranial nerve functions is defined by the possibilities of their anatomical identification during the microsurgical procedures.[14,15,17,23,24,28–31] Radiosurgery is a modality of treatment of skull base tumors, which avoids the direct approach to vessels and nervous structures.[6,7,13,36]

The application of SF during cranial base surgical approaches was an extension of the previous uses of such substance. The grade of the skull base tumor enhancement by the SF was strongly positive. Even with the standard white-light microscope illumination, the enhancement of the tumors was evidenced by the yellow pigmentation after injection of the SF [Figures 1 and 2].

The purpose of the study was to test the hypothesis of skull base tumor enhancement by SF at the operative field. The inclusion of four histological different tumors was carried out to develop the possible application in forthcoming studies, considering this first description. Nevertheless, the enhancement differences among different histological subtypes and the clinical impact of the SF application in the skull base lesions should be evaluated with other specific design studies.

In the present series, the use of the 1 g of SF 20% was the same as that of the original description by Moore *et al*.[20] The authors used such a dose due to the fact that in the former study they used the SF in different histological subtypes beyond gliomas. In gliomas series, the usual dose is 20 mg/kg.

Moore *et al*. described that the maximum tissue fluorescence occurred in about 2 hours after injection of the SF and the effect persisted for at least 5 hours.[20] In the skull base lesions presented, the dye was evident after 10 minutes of SF injection and persisted during tumor dissection for several hours, which is consistent with the initial observation of fluorescence in tumors located at different sites.

The dissections were performed during the time between pre- and post-injection pictures, in order to avoid prolonging surgical time. This explains some differences observed in the pictures of Figure 1.

SF staining during glioma surgery is probably related to blood brain barrier (BBB) disruption. It is discussed that BBB disruption plays the principal role for the gadolinium enhancement of the tumors on magnetic resonance imaging (MRI).[32] Tumors included in this preliminary study present marked enhancement by gadolinium on MRI and, such an aspect could be an explanation for the strong SF capture by the tumors. However, SF is a high molecular substance and in tumors with intact BBB, the dye could remain intravascular and affect tumor enhancement.

The evidence of SF in the surgical cavity helped the radical removal of gliomas since the first description.[20] The contemporary studies also reveal superior total resection results based on the same aspect.[32,35] These previous evidences point to one possible application of the SF in the cranial base neoplasms, which is the identification of some residual tumors after initial resection. In the clinoidal meningioma illustrated, Figure 2b shows that SF seems to enhance the dura around the tumor. Such data could represent dural extension of the tumor.

The use of SF is very simple. The substance presents a low cost, safe, and universally available option. The method described does not require any special microscope. In fact, it can be reproduced in any department, using a standard white-light microscope.

## CONCLUSIONS

The enhancement of the tumors by the SF in the series was consistent. The highly positive effect observed in the present study allows to question about the possibility of future application of SF as an adjuvant tool for skull base surgery.
